# Peptides derived from gp43, the most antigenic protein from *Paracoccidioides brasiliensis,* form amyloid fibrils in vitro: implications for vaccine development

**DOI:** 10.1038/s41598-021-02898-5

**Published:** 2021-12-06

**Authors:** Thyago R. Cardim-Pires, Ricardo Sant’Anna, Debora Foguel

**Affiliations:** Instituto de Bioquímica Médica Leopoldo de Meis, Universidade Federal do Rio de Janeiro - Centro de Ciências da Saúde, Av. Carlos Chagas Filho 373, Bloco E, sala 42, Ilha do Fundão, Rio de Janeiro, RJ CEP 21941-902 Brazil

**Keywords:** Peptides, Protein folding

## Abstract

Fungal infection is an important health problem in Latin America, and in Brazil in particular. *Paracoccidioides* (mainly *P. brasiliensis* and *P. lutzii*) is responsible for paracoccidioidomycosis, a disease that affects mainly the lungs. The glycoprotein gp43 is involved in fungi adhesion to epithelial cells, which makes this protein an interesting target of study*.* A specific stretch of 15 amino acids that spans the region 181–195 (named P10) of gp43 is an important epitope of gp43 that is being envisioned as a vaccine candidate. Here we show that synthetic P10 forms typical amyloid aggregates in solution in very short times, a property that could hamper vaccine development. Seeds obtained by fragmentation of P10 fibrils were able to induce the aggregation of P4, but not P23, two other peptides derived from gp43. In silico analysis revealed several regions within the P10 sequence that can form amyloid with steric zipper architecture. Besides, in-silico proteolysis studies with gp43 revealed that aggregation-prone, P10-like peptides could be generated by several proteases, which suggests that P10 could be formed under physiological conditions. Considering our data in the context of a potential vaccine development, we redesigned the sequence of P10, maintaining the antigenic region (HTLAIR), but drastically reducing its aggregation propensity.

## Introduction

Fungal infection is an important public-health problem afflicting more than a billion people and contributing to the death of 1–2% of the patients. Mycoses are especially important in Latin America, and in Brazil in particular (80% of the cases), where the climate favors yeast proliferation and infection, prevailing in rural workers of endemic areas. *Paracoccidioides* is the genus of fungi responsible for paracoccidioidomycosis (PCM). Among its five species are *P. brasiliensis* (Pb) and *P. lutzii*. The lungs are the primary infection site, but oral mucosa and airways can also be affected^1^.

The success of yeast-host interaction depends on several regulatory mechanisms as well as the expression of several different virulence factors. Among these are adhesins, which are surface proteins that recognize extracellular matrix (ECM) components of the host cells^2,3^.

**Figure 1:**
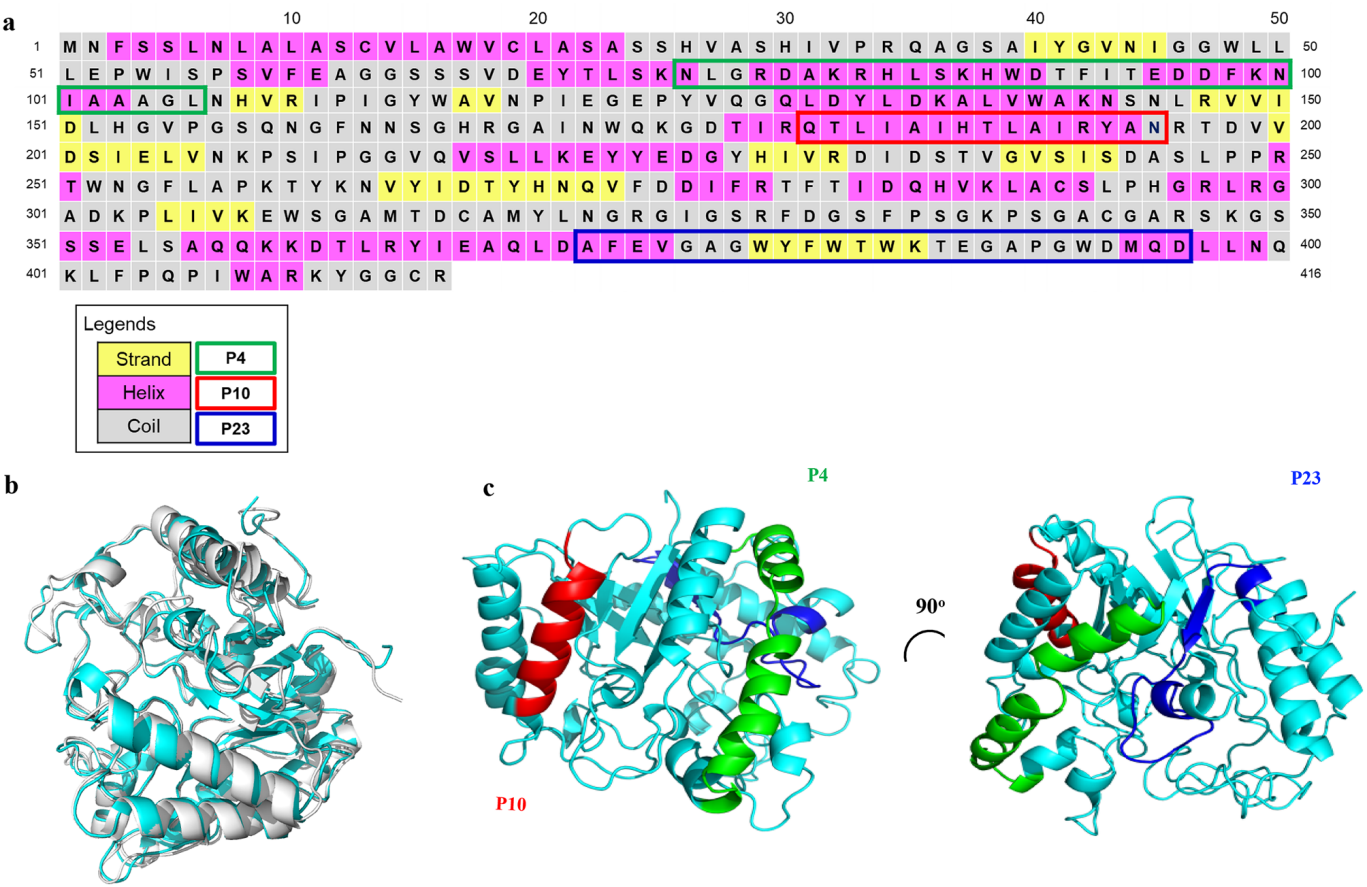
Primary sequence and predicted secondary structure elements of gp43 from *Paracoccidioides brasiliensis* strain B339 highlighting the three peptides studied here (P4, P10 and P23). (**a**) The primary structure was obtained at GenBank access number AAG36697.1. Secondary structure elements of gp43 were predicted by PSIPRED and are indicated by pink, yellow and grey cells where color refers to, respectively, α-helix, β-strand and random-coil structures. Green, red and blue outlines represent P4, P10 and P23 primary sequences, respectively. (**b**) The structural model of gp43 was generated by using AlphaFold software (cyan), which was aligned with the β-glucanase structure (gray). (**c**) Positions of P4, P10 and P23 in the structural model of gp43 are depicted in green, red and blue, respectively. Superposition of both structures in B was generated by PyMOL (The PyMOL Molecular Graphics System, Version 1.2r3pre, Schrödinger, LLC.).

Gp43 is a secreted glycoprotein involved in fungal adhesion to epithelial cells and macrophages and is the most studied protein of *Paracoccidioides brasiliensis,* although other adhesins (moonlighting proteins) are also important to fungal virulence^2,4^. Gp43 contains regions that drive interactions with laminin, collagen and fibronectin, allowing Pb adhesion to the cell^5^. Gp43 has 416 amino acids, with secondary and tertiary structures not yet described. It presents high sequence homology (80% similarity) to the yeast exo-1,3-β-glucanase (UniProtKB—C1H4T0 Fig. 1a), but no detected enzymatic activity^6^ since the catalytic site differs significantly in the Pb protein. Secreted during fungal infection, gp43 is the main antigen detected in patients^7^ and it contains epitopes capable of eliciting a cellular immune response in animal models and in human patients leading to the production of IFN-γ by lymphocytes, which stimulates the formation of granulomas to contain the yeast cells^8,9^.

Since gp43 is the main PCM diagnostic antigen containing epitopes that elicit delayed-type hypersensitivity in animals and humans, in 1998, Taborda and coworkers synthesized 25 gp43-derived peptides to identify the T-cell epitopes. From this extensive study, the authors identified P10 (QTLIAIHTLAIRYAN), a specific stretch of 15 amino acids that spans the region 181–195 and was recognized by T lymphocytes in peripheral blood mononuclear cells of mice and humans^10,11^. Mice immunized with P10 developed lung infection 200 times less intense than the unimmunized mice, a response that was as effective as immunization with full-length gp43.

Later, Iwai and coworkers (2003) used an in-silico approach (TEPITOPE algorithm)^12^ to localize peptide sequences from gp43 that would most likely bind multiple human leucocyte antigen-DR (HLA-DR) molecules and tested their recognition by T-cells from sensitized individuals. The most recognized peptide from peripheral blood mononuclear cells from PCM patients was gp43 (180–194), recognized by T-cells from 53% of patients. This region of gp43 is a close match of P10. Several groups are studying P10 as a candidate for a vaccine against PCM and evaluating different adjuvants to potentiate the immunological response^13^. These studies include the use of a DNA vaccine encoding the P10 sequence^14^. Magalhães and collaborators showed that dendritic cells primed with P10 protect the host against the development of mycosis and these cells are also effective in the treatment of well-established infections^8^.

The use of synthetic peptides to develop vaccines against microbes has been envisioned for a long time^15^. However, little is known about the biophysical properties of these peptides in solution, such as their tendency to aggregate into insoluble material, including amyloid fibrils. This latter type of aggregate is involved in amyloid diseases and small pieces of amyloid fibril can seed the aggregation of other cellular proteins, something unwanted for vaccine development^16^.

Here, we present evidence that synthetic P10 forms typical amyloid aggregates in solution in very short times (a few seconds). In-silico analyses using different algorithms revealed that gp43-derived peptides, including P10, have high aggregation propensity. Molecular modeling approaches showed that several stretches of the P10 sequence fit well into structural models of steric zippers, ensembles present in highly organized amyloid aggregates^17^. Interestingly, seeds formed by mechanical fragmentation of amyloid fibrils composed of P10 were able to induce the aggregation of a non-aggregating peptide also derived from gp43, named P4 (NLGRDAKRHLSKHWDTFITEDDFKNIAAAGL), but not the peptide P23 (AFEVGAGWYFWTWKTEGAPGWDMQD). Mass spectrometry showed that the fibrils composed of P4 seeded by P10 contains both sequences, suggesting a co-aggregational mechanism. P4 and P23 are potent modulators of local and systemic inflammation^9^ since they inhibit phagocytosis of zymosan particles and Pb yeast by macrophages. Finally, in-silico proteolysis studies with gp43 revealed that aggregation-prone, P10-like peptides could be generated by the action of several enzymes including proteinase K, trypsin and pepsin. Besides host endogenous proteases, the levels of proteases secreted by Pb, including an aspartyl proteinase (pepsin-like), are increased upon infection^18^, so we envision that P10 or a P10-like peptide may be formed under physiological conditions, giving rise to amyloid fibrils. Altogether these data call attention to the fact that candidate peptides for vaccine development can aggregate in solution with important implications for their efficacy and safety.

## Results

### Structural model and prediction of aggregation propensity of gp43 and its derived peptides

Figure 1a shows the primary sequence of gp43 (416 amino acids), highlighting in colored boxes the positions of the three peptides studied here, namely, P4 (green box), P10 (red box) and P23 (blue box). Since the tridimensional structure of gp43 has not been solved yet, its elements of secondary structure were predicted by using the program PSIPRED^19^ and are depicted in this panel. The protein is predicted to be mainly composed of α-helices (13; marked in pink) with small β-strands (8; marked in yellow) and random-coiled/unstructured regions (marked in gray) intercalated among them. Gp43 has a primary sequence with high similarity to yeast β-glucanase (glucan 1,3-β-glucosidase). Since the high-resolution structure of β-glucanase has already been solved (PDB1CZ1)^20^, we ran PSIPRED with β-glucanase as a control and the algorithm retrieved with great accuracy the known pattern of secondary structure elements of the enzyme (prediction: 14 α-helices and 8 β-strands; x-ray crystallography: 15 α-helices and 8 β-strands).

In the absence of a 3D structure of gp43, we built an in-silico model with AlphaFold^21^. AlphaFold is a state-of-the-art neural network-based methodology able to predict high resolution protein structures. It is the first computational approach demonstrating accuracy competitive with experimental structures in most cases and greatly outperforming other methods. Figure 1b displays the model of gp43 superposed on the structure of β-glucanase showing small deviations, most of them in loops/unstructured regions (RMSD of the alignment is 1.195 Å). We also performed the modeling with the program RaptorX^22,23^ and obtained almost complete superposition with the β-glucanase structure (not shown).

Figure 1c shows in the generated model of gp43 the position of the three peptides studied here: P4 (green), P10 (red) and P23 (blue). As seen (Fig. 1a and c), the 31 amino acids of P4 are predicted to span an α-helix-rich region of the protein (amino acids 76–106), as well as the 15 amino acids of P10 (amino acids 181–195). Peptide P23, with its 25 amino acids (amino acids 372–396), however, encompasses a region supposedly devoid of a defined secondary structure with a small β-strand in its middle. As seen in panel c, while P4 and P10 are located on the surface, P23 is mainly buried in the core of the protein.

As mentioned before, these three peptides from gp43 are highly immunogenic, especially P10, which has been envisioned as an element in the development of a vaccine against Pb^8,24^. As it is evident in the structural model of gp43 (Fig. 1b and c) and according to the predictor of solvent accessibility NetSurfP^25^, a little more than half (8 out of 15) residues of P10 are considered highly exposed. These residues are Q181, I184, H187, T188, I191, R192, A194 and N195. This high degree of exposure is potentially incompatible with the high hydrophobicity of the peptide. The Grand Average of Hydropathicity (GRAVY) of P10 is 0.607, while that of Aβ peptide, which partially spans the cell membrane and is involved in Alzheimer’s Disease^26^, is 0.205 (ProtParam Expasy; 27). This latter feature led us to consider whether P10, when in solution, might tend to aggregate, and this property should be evaluated before using it for vaccine development.

**Figure 2:**
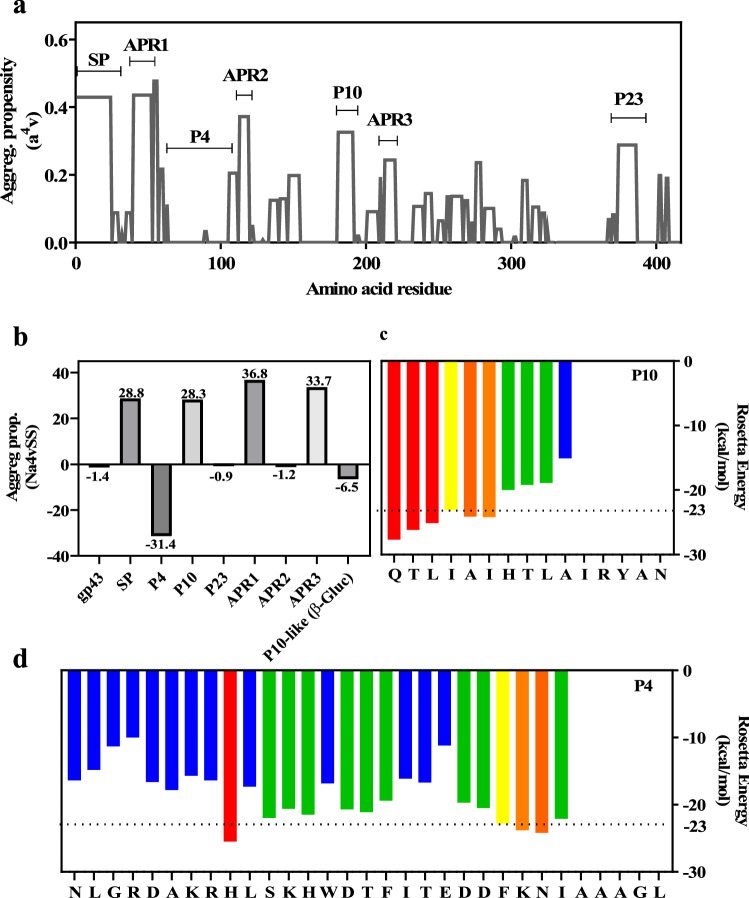
Aggregation propensity analysis of entire gp43 and its derived peptides. (**a**) Aggrescan analysis of entire gp43 showing its aggregation propensity scores as a function of its primary sequence. SP: signal peptide (amino acids 1–35); APR1, 2 and 3: amyloid-prone regions 1, 2 and 3. Regions corresponding to P4, P10 and P23 are marked. (**b**) Aggrescan analysis of different segments of gp43 (SP, APR, P4, P10 and P23) as well as the corresponding sequence of P10 in β-glucanase from *P. lutzii* (QTLAAIRALANRYAK). (**c** and **d**) ZipperDB analysis of P10 and P4, respectively, showing the hexapeptides predicted to form steric zippers (Rosetta energy values bellow − 23 kcal/mol). Yellow, orange and red bars indicate the first residue of a hit hexapeptide (N to C terminal); green and blue bars indicate that the hexapeptide beginning at that residue does not fit into a steric zipper.

In order to sort out the aggregation propensity of the different segments of gp43, including the P10 region, Aggrescan was employed^27^. Aggrescan is a well-validated algorithm that allows the identification and evaluation of aggregation-prone regions (APRs) within proteins and peptides. The Aggrescan data analysis for full gp43 is depicted in Fig. 2a.

In Fig. 2a, the regions corresponding to the three peptides of gp43 studied here are marked. As seen, P10 and P23 are inserted in regions with high aggregation propensity, while P4 is not. There are other regions dispersed along the protein with high aggregation propensities (those with high scores are labelled APR 1–3), including the region encompassing the signal peptide (SP) at the N-terminal region (~ the first 50 residues).

Figure 2b depicts the aggregation propensities of the peptides under study, which are compared to the aggregation potential of the entire gp43 as well as with other APRs present in the protein. Overall, the whole gp43 protein has no tendency to aggregate. This is also true of P4 and P23, even though the latter spans a region of gp43 with high aggregation scores (panel A): when analyzed in isolation and normalized to its number of residues, it does not have a high aggregation score. On the other hand, P10 is among the APRs with the higher aggregation propensity scores, like the signal peptide located at the N-terminal region of the protein (N-term 1–35). Interestingly, the region corresponding to P10 in β-glucanase from *Paracoccidioides lutzii* (QTLAAI**R**ALAN**R**YA**K**) has no tendency to aggregate according to Aggrescan (Fig. 2b; P10-like), probably due to the presence of positively charged residues (marked in bold), two in the middle (R7 and R12) and one at the end (K15) of the peptide, which might act as aggregational gatekeepers^28–30^ . P10 in gp43 (QTLIAIHTLAI**R**YAN) has only one arginine (position 12). These observations have important implications and will be discussed below.

To support our view, we also used the consensus aggregation predictor method Amylpred2, which is based on the combination and weighting of the outputs of several predictors into one single output. This method assembles the different concepts behind each predictor to increase robustness and reduce method-specific bias^31^. Amylpred2 did not detect any consensus APR in P4. On the other hand, in P10, it detected the presence of an APR encompassing almost the entire peptide sequence (residues 2–13; 80% of the sequence). Regarding the peptide P23, Amylpred2 pointed out a short region spanning from residues 6–13 with aggregation propensity (32% of the peptide sequence). In P23, position 14 is occupied by a lysine, which may act as an aggregational gatekeeper.

Next, ZipperDB^32^, an algorithm that predicts the ability of a peptide to form amyloid steric zippers, was employed with the three peptides of interest and the data are presented in Fig. 2c (P10) and D (P4). The 3D-profiling method of ZipperDB was the first amyloid predictor built upon 3D structural data. At the core of amyloid fibrils is the cross-β spine, a long stretch of β-sheets formed by the constituent protein or peptide^33^. In an amyloid steric zipper, the side chains from two β-strands form a tightly interdigitating dehydrated interface, so that the resulting β-sheet bilayer forms the fundamental building block of the fibrillar aggregates^33^. In the predictor approach, each hexapeptide from the query sequence is threaded onto the experimentally determined 3D-structure of the NNQQNY peptide, and the energetic fit is evaluated by using the RosettaDesign energy function^34^.

According to the data presented in Fig. 2c, in P10 there are several hexapeptides able to adopt an amyloid steric zipper structure, as evidenced by consecutive hot-colored bars (yellow–red) in its N-terminal region, where it reaches the threshold value of -23 kcal/mol. In the graph, the Rosetta energy value of each bar corresponds to the hexapeptide beginning at that position and running + 5 residues to the right. P4 also contains 4 non-contiguous hexapeptides able to form zippers, three of them in its C-terminus and one in the middle of its sequence (Fig. 2d). P23 does not contain any region in its structure that can be accommodated in a steric zipper structure (not shown).

**Figure 3:**
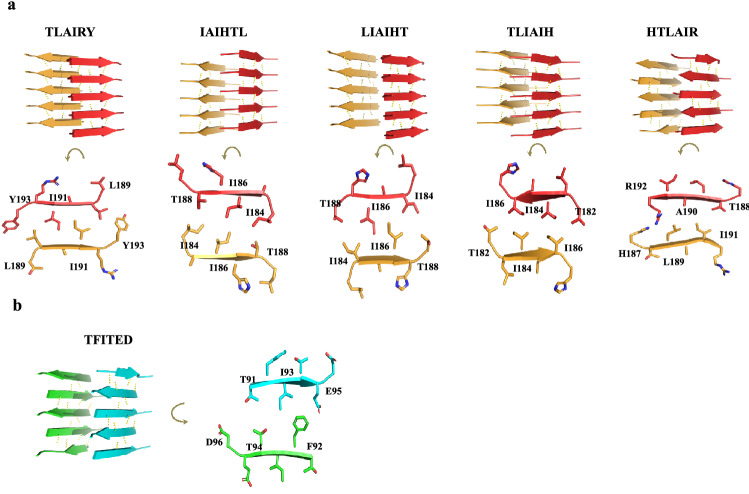
Hexapeptides derived from P10 and P4 (only a single stretch) form steric zippers in silico. P10 (**a**) and P4 (**b**) sequences were analyzed by Cordax (LOURO et al., 2020). The algorithm identified five hexapeptides in the sequence of P10 (TLIAIH, LIAIHT, IAIHTL, TLAIRY and HTLAIR) capable of forming steric zippers. Except for HTLAIR, each peptide forms two sheets (orange and red) composed of parallel β-strands (antiparallel in HTLAIR). In panel (**a**), the lower images show details of the interdigitation of the lateral chains of the amino acids in the steric zippers facing the interior of the bilayer. (**b**) Only one hexapeptide of P4 (TFITED; blue and green) adopts a steric zipper structure in an antiparallel fashion.

Since ZipperDB identified several hexapeptides capable of forming amyloid steric zippers, we used the recently developed program Cordax^35^ to build structural models of the putative amyloid zippers present in P10 (Fig. 3a) and P4 (Fig. 3b). Interestingly, Cordax detected the presence of 5 hexapeptides capable of self-interacting to form steric zippers as amyloid cores in the P10 sequence. As seen in the models presented in Fig. 3a (upper images), TLAIRY, IAIHTL, LIAIHT, TLIAIH are supposed to form zippers composed of parallel β-sheets (sheets presented in yellow or red), while HTLAIR adopted an anti-parallel β-sheet topology. As mentioned before, in a steric zipper structure, the side chains of the residues in one strand placed in one side of the sheet face the side chains of residues in the strands belonging to the complementary sheet, creating the bilayer of the amyloid steric zipper. Figure 3a (lower images) presents the details of this side-chain complementarity observed in the zippers formed by P10.

Regarding P4, only a hexapeptide (TFITED) in the central region of the sequence can form an amyloid steric zipper (Fig. 3b) in an anti-parallel architecture. As seen in the primary sequence of P4, out of 31 residues, 10 are charged (NLG**RD**A**KR**HLS**K**HW**DTFITEDD**F**K**NIAAAGL, excluding histidine) and are dispersed along its sequence, and these charges probably hinder zipper formation. The two charges of TFIT**ED** are contiguous and present at the C-terminus of the peptide. As seen in the predicted model of this steric zipper (panel B, right), these two negative charges lie at opposite ends of the zipper, facing TF residues. In line with ZipperDB predictions, Cordax did not detect hexapeptides capable of forming amyloid steric zippers in the sequence of P23.

Taken together, these bioinformatics analyses indicate that P10, the peptide with the highest antigenic properties of gp43, presents high aggregation propensity, being able to form amyloid steric zippers at least in silico, a property that should be considered as it is a candidate for vaccine development. The next experiments were performed in order to determine whether or not these peptides aggregate in solution.

### P10 forms amyloid fibrils in aqueous solution at neutral pH and seeds the aggregation of P4

**Figure 4:**
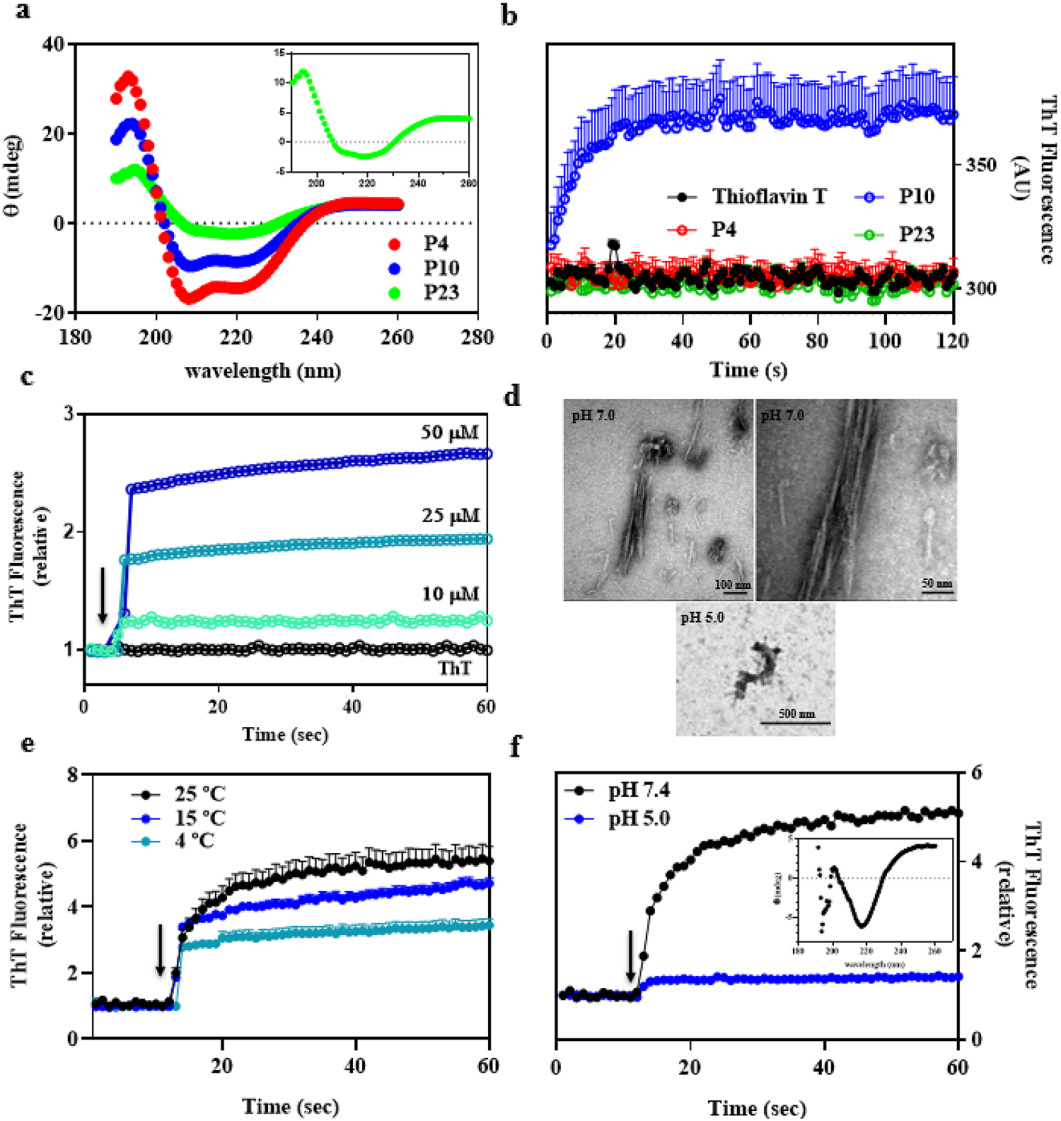
In solution, P10 undergoes aggregation forming amyloid fibrils. (**a**) Circular dichroism spectra of 100 μM of soluble P4, P10 and P23 in 5% TFE (see also the inset for more details of P23 secondary structure). (**b**) Kinetics of P10 aggregation (blue) at pH 7.4, 25 °C by measuring Thioflavin-T (ThT) binding. P4 and P23 do not form ThT-positive aggregates in solution under these conditions. In all experiments (except in panel 4C), the concentrations of peptides were 100 μM. (**c**) Aggregation of P10 (pH 7.4; 25 °C) shows concentration dependence. [P10] = 10, 25 and 50 μM, as indicated. (**d**) TEM images show the presence of mature amyloid fibrils composed of P10 only at pH 7.4 (25 °C), while at pH 5.0 amorphous aggregates predominate; the right-hand image at pH 7.4 is a zoom of the left-hand one. The inset of panel (**f**) shows the circular dichroism spectrum of the amyloid fibrils composed of P10, showing their β-sheet content. (**e**) Aggregation of P10 at pH 7.4 (100 μM) diminishes at low temperatures: Kinetics were performed at 4, 15 and 25 °C, as indicated. (**f**) Aggregation of P10 at 25 °C, 100 μM at pH 5.0 (blue) and 7.4 (black). The arrows in panels C, E and F indicate the moment when the peptides were added to the buffer. ThT binding was measured by exciting the samples at 450 nm and recording emission at 485 nm. Error bars in (**b**) and (**e**) are SD in three independent experiments. In (**c**), (**e**) and (**f**) the error bars are shorter than the size of the symbols.

Since in-silico approaches indicate that at least P10 has APRs, *in-vitro* studies were carried out to evaluate their aggregation properties. In order to evaluate the secondary structures of P4, P10 and P23 in solution before aggregation, circular dichroism measurements were performed. The solvent TFE had to be used to keep the peptides soluble. As seen in Fig. 4a, P10 and P4, when dissolved in TFE, assume α-helix-rich structures, while P23 has a spectrum more closely related to a β-sheet-rich peptide (Fig. 4a, inset). This secondary-structure data to some extent recapitulates what has been predicted from the gp43 structure, when it was modeled onto the structure of β-glucanase (Fig. 1). Accordingly, in this model, P10 and P4 encompass regions of the protein rich in α-helices, while P23 seems to be in a region of the protein devoid of secondary-structure elements with only a small β-strand in it.

Next, the peptides (100 μM) were incubated in aqueous solution (pH 7.4, 25 °C) in the presence of Thioflavin-T (Th-T), a specific probe for amyloid fibrils. Figure 4b shows that P10 forms Th-T-positive aggregates in ~ 10 s, which is suggestive of their amyloidal nature, while P4 and P23 did not. The next set of experiments was performed with P10, since this was the only peptide able to aggregate in solution, in agreement with most of the predictions (Figs. 2 and 3). Figure 4c shows the aggregation profiles at pH 7.4, 25 °C of P10 at 25, 50 and 100 μM as measured by Th-T and light scattering (not shown), confirming the amyloidal aggregation of this peptide in very short times, as well as its concentration dependency. Images of the aggregates formed are depicted in panel D, where it is possible to see mature amyloid fibrils, which present the typical β-sheet-rich structure measured by circular dichroism (inset of panel F). To better characterize whether P23 would form amyloid fibril in solution, TEM images were performed, and no fibrils were observed (not shown).

Figure 4e shows that P10 amyloid aggregation is diminished at low temperatures (pH 7.4), as observed in other aggregation processes^36^.

Since the aggregation of proteins and peptides is influenced by pH and the endosomal compartments, where antigens are processed and docked at the MHC II cleft are acidic, we evaluated the aggregation of P10 at pH 5.0. Figure 4f shows that P10 behaves differently at pH 5.0 and its aggregation was almost completely abolished. TEM images of the species formed at pH 5.0 did not show any mature amyloid fibrils as easily seen at pH 7.4, but only amorphous aggregates (Fig. 4d). These data are interesting and suggest that in endosomal compartments, aggregation might be prevented.

Next, we asked whether aggregation of P10 would occur in complete or incomplete Freund adjuvants, the vehicle used to produce vaccines. Interestingly, when dissolved in adjuvants at 100 μM, 25 °C (pH ~ 6.5), P10 did not undergo aggregation. Instead, it remained soluble, as measured by ThT binding (Supplementary Fig. S1).

**Figure 5:**
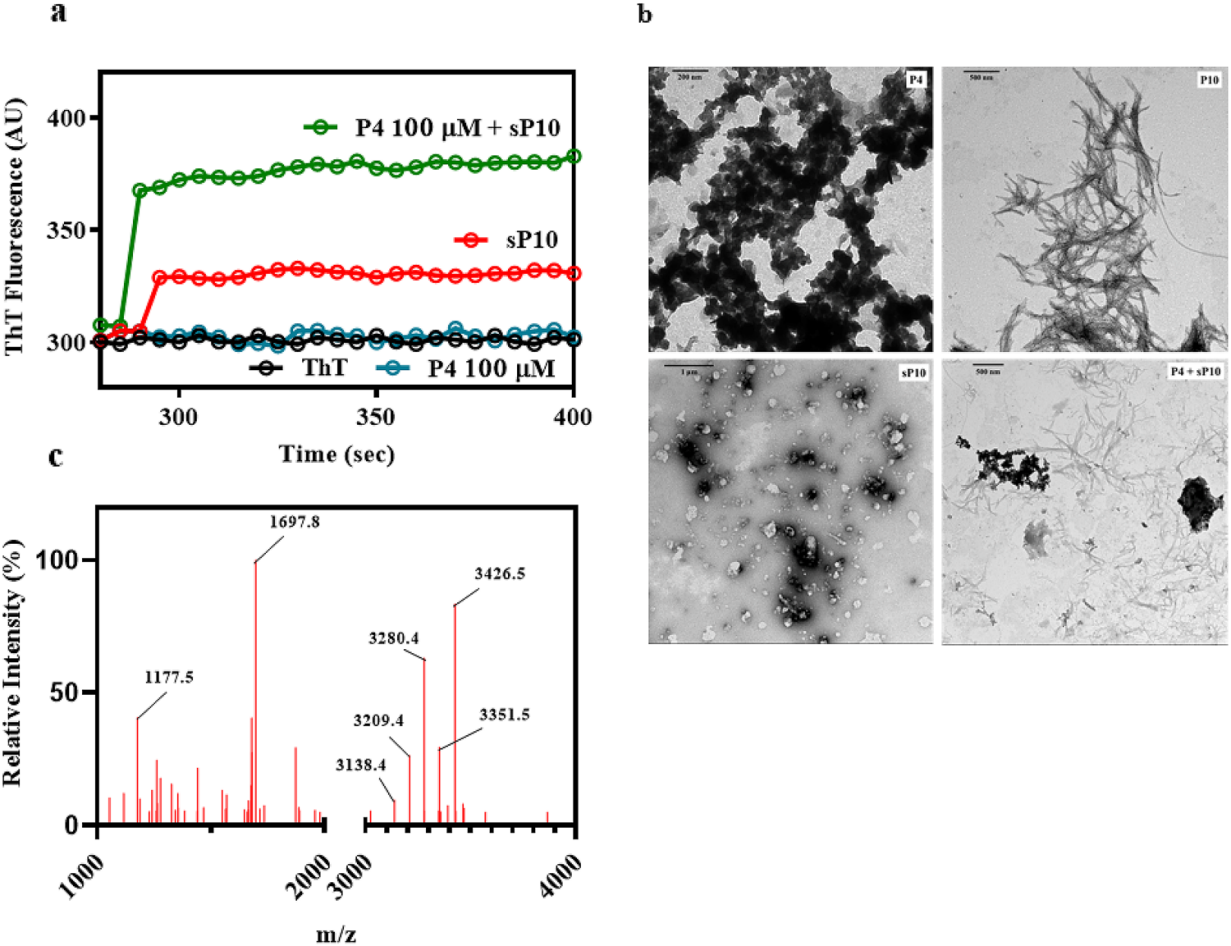
Seeds composed of P10 (sP10) can seed P4 amyloidal aggregation. (**a**) P4 alone (blue, 100 μM) does not aggregate in solution unless P10 seeds are added (green; 5% seeds). In red is shown the ThT signal of the seeds alone in solution and in black is the ThT emission when free in solution. (**b**) TEM images of the amorphous aggregates formed by P4 alone (P4, upper left), P10 fibrils (P10, upper right), seeds of P10 (sP10; lower left) and aggregates formed when P4 is seeded by P10 (P4 + sP10; lower right). (**c**) Mass spectrometry analysis of P4 fibrils grown in the presence of P10 seeds showing the expected molecular masses of P10 (mass: 1,697) and P4 (mass: 3,426). These data suggest the incorporation of P4 into the P10 seeds giving rise to the appearance of hybrid fibrils. All experiments were performed at pH 7.4, 25 °C.

Amyloid formation can be accelerated by the presence of small fragments of mature amyloid fibrils, called seeds^37,38^. We asked whether seeds derived from P10 fibrils (sP10) would be able to seed the aggregation of P4 and P23. Interestingly, the addition of 5% seeds of P10 to a solution with 100 μM P4 (pH 7.4, 25 °C), which does not aggregate by itself, was able to induce immediately the aggregation of P4 as seen by Th-T binding (Fig. 5a, green curve) and EM imaging (Fig. 5b; P4 + sP10). Similar experiments were tried with P23, but this peptide forms a viscous solution when diluted in aqueous buffer, which makes this type of experiment unfeasible. This result suggests that seeds composed of P10 catalyze the aggregation of P4, even though P4 does not tend to aggregate in solution. It may be that the steric-zipper architecture predicted for P10 fibrils catalyzes the aggregation of P4 through the unique region of P4 with propensity to form zippers (TFITED; Fig. 3b).

In order to confirm that seeds of P10 were indeed seeding the aggregation of P4, amyloid fibrils were collected by centrifugation from the seeding experiments, washed, resuspended in 9 M urea for their complete dissociation and analyzed by mass spectrometry (Fig. 5c). As seen, a peptide with the molecular mass of P4 (3,426.5) was present within these fibrils, as well as a peptide with the expected P10 mass (1,697.8). Other molecular masses detected were compatible with P4 and P10 degradation products (P4: 3,351.5; 3,280.4; 3,209.4 and 3,138.4; P10: 1,177.5).

### Aggregation propensity of P10-like peptides derived from gp43 proteolysis: in-silico analysis

Another important feature to be considered is that gp43 is secreted from the yeast cells during infection, thereby being exposed to several proteases from the host. Besides, although in lower quantities then other secreted proteins, the levels of gp43, together with GAPDH and aspartyl proteinase, are increased and these proteins secreted during yeast biofilm formation^2^. This latter protease may also target gp43, fragmenting this protein into small pieces. Thus, we examined whether gp43 could be processed by different proteases by using the program ExPASy PeptideCutter^39^. Table 1 presents the result of these analyses showing only the primary sequence of the peptides in which P10 or P10-like peptides are formed. As seen, proteinase K digestion of gp43 is able to release the peptides R-P10 (P10 with an extra R at the N-terminal) and R-P10(-2) (the same as R-P10 shortened by two residues at the C-terminal), while trypsin generates (+5)P10 + R (P10 with five extra residues at the N-terminal and R at the C-terminal) and P10 + R (P10 with an extra R at the C-terminal). Pepsin, which is an aspartic protease of the same family as Pb protease, also gave interesting results and the fragments generated by its enzymatic activity are (+7)P10(−2) (P10 with seven residues at the N-terminal shortened two residues at the C-terminal) and (−3)P10(−2) (P10 shortened three residues at the N-terminal and shortened two residues at the C-terminal). In all cases, the cleavage products contain the APR present in the P10 sequence.

**Table 1:**
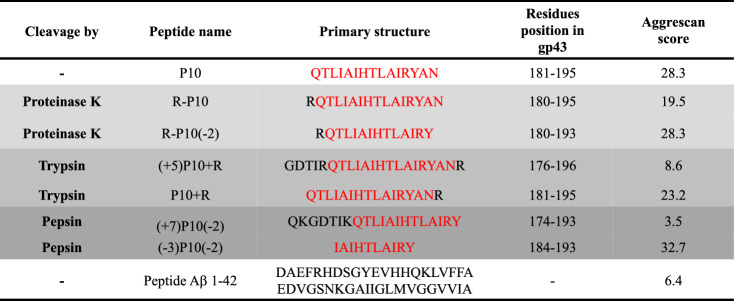
In-silico enzymatic digestion of gp43 shows that P10-like peptides can be formed.

Full gp43 primary sequence was subjected to the ExPASy PeptideCutter tool to identify possible cleavage sites. Proteinase K, trypsin and pepsin were the enzymes predicted to cleave gp43, generating P10-like peptides, while conserving P10 core sequence (HTLAIR). The peptide sequences and their positions in gp43 are depicted in the Table, where: R-P10 = P10 with an extra R at the N-terminal; R-P10(−2) = the same as R-P10 shortened two residues at the C-terminal; (+5)P10 + R = P10 with five extra residues at the N-terminal and R at the C-terminal; P10 + R = P10 with an extra R at the C-terminal; (+7)P10(−2) = P10 with seven residues at the N-terminal and shortened two residues at the C-terminal, and (−3)P10(−2) = P10 shortened three residues at the N-terminal and two residues at the C-terminal. In all sequences, P10 is colored in red. Aggrescan was used to estimate the aggregation propensities of the peptides. Peptide Aβ 1–42 was included for comparison.

In the last column of Table 1 are presented the amyloidogenic propensities of all peptides derived from gp43 digested with proteinase K, trypsin and pepsin calculated by Aggrescan. Aggrescan shows that P10 and all P10-like peptides derived from in-silico enzymatic digestion of gp43 exhibit substantial aggregation propensity scores, even higher than Aβ 1–42 (Aggregation propensity score = 6.4). Exceptions to this behavior are (+5)P10 + R and (+7)P10(−2), which display much lower aggregation propensities than the other products of digestion. This indicates that the additional residues inserted at the N-terminal of P10 diminish its aggregation propensity, probably because these short extensions include charged residues, R, K and D, which display gatekeeper properties preventing their aggregation: (+5)P10 + R contains one D and one R at its N-terminal extension, plus an additional R at the C-terminus, while (+7)P10(−2) contains two K and one D at its N-terminal extension.

Interestingly, the shortest of the peptides, namely (−3)P10(−2), generated by pepsin digestion, has the highest aggregation propensity among them all. This short peptide encompasses the sequence HTLAIR, the essential region of the epitope^40,41^, which, as shown before (Fig. 3), forms an anti-parallel steric zipper. Besides, it has only one charged residue in its N-terminal extremity, which probably diminishes its solubility.

## Discussion

Peptide-based vaccines are promising, and several studies and clinical trials illustrate this application (reviewed by 42), including in COVID-19, where peptides derived from SARS-CoV-2 are being investigated for this purpose^42^. Indeed, there are studies in phase 1, 2 and 3 aiming to use peptides derived from different pathogens as vaccine candidates^43,44^.

Certainly, the initial search for a candidate peptide for vaccine development must be based on its immunogenic potential. A possible initial strategy is the use of algorithms to help in the identification of putative epitopes in the primary sequence of a given protein capable of inducing a positive, desirable T-cell and B-cell immune response^45^. Once identified, these epitopes must be chemically synthesized as peptides for in-vitro and in-vivo studies, when its effective cellular and humoral responses must be evaluated as well as its protective potentials, when the host is challenged by the real pathogen. The choice of an adequate adjuvant is another important aspect of the process, due to the low capacity of peptides to induce innate immunity and a specific adaptative immune response, when administered alone.

However, the physicochemical properties of a candidate peptide must also be considered including its aggregation propensity when in solution, since this aggregation could undermine its future use as vaccine.

Here we studied the aggregation potential of three peptides derived from gp43 from Pb, one of which, P10, is of special importance, since it has been considered as a candidate for vaccine development. As shown here by different methodologies, P10 aggregates almost instantaneously into amyloid fibrils when in solution at pH 7.4 and less extensively at pH 5.0 (Fig. 4). These fibrils exhibited the same architecture as that observed in amyloid fibrils present in amyloidogenic diseases^17^. Although adjuvants inhibited the aggregation of P10 (Supplementary Fig. S1), we do not know what happens when the peptide in adjuvant reaches the plasma, which needs to be explored in the future. The other two peptides, namely P4 and P23, did not form typical amyloid fibrils when in solution, although aggregates were observed with the former. Interestingly, in the presence of seeds composed of P10, P4 formed amyloid fibrils as seen by ThT binding, TEM and MS (Fig. 5).

Bioinformatics studies with P10 indicate that there are regions within this peptide capable of forming steric zippers (Fig. 3a). According to a classification proposed by Eisenberg and co-workers^17^ these zippers can be classified into eight types of topologies. These topologies are distinguished by whether the strands in the sheets are parallel or antiparallel, whether the sheets pack with the same (‘face-to-face’) or different (‘face-to-back’) surfaces to one another forming the zipper, and whether the sheets are oriented parallel (‘up–up’) or antiparallel (‘up–down’) with respect to one another. Peptides TLIAIH, LIAIHT, IAIHTL and TLAIRY form type 1 zippers, where the strands are parallel in the same sheet, but the sheets are anti-parallel with respect to one another (up-down) and the sidechains of similar residues pair face-to-face (Fig. 3a). Interestingly, the zipper formed by the hexapeptide HTLAIR falls into class 7, where the strands on the sheets are antiparallel and they pack together face-to-back, where the zipper is formed by pairing of sidechains of different residues of the sequence (Fig. 3a). Regarding P4, it possesses only one hexapeptide stretch (TFITED) able to pack as a zipper with class 7 topology (Fig. 3b). It is possible that the seeded aggregation of P4 by P10 seeds is nucleated around this only sequence of P4 able to adjust into a steric zipper.

Curiously, there is an old study reported in a thesis from Brazil which describes that Pb infection triggers amyloid deposition in hamster kidney, an event that affects the animal’s renal function. These data, although not explored at that time by the research group, suggests that aggregation can occur upon Pb infection^46^.

Another interesting property of P10 aggregation is its dependency on neutral pH (Fig. 4f). There are interesting studies proposing that aggregation of antigens might be a strategy of phagocytic cells to concentrate and preserve the integrity of these antigenic peptides before their insertion into the MHC-II cleft and displacement of CLIP from the cleft^47^. Thus P10, when cleaved from gp43 during antigen processing in early/late endosomes (pH > 5), might form aggregates very fast inside these compartments until the pH is acidified by the fusion with lysosomes, generating the endolysosomes (pH < 5). This brings about the dissociation of the peptides from the aggregates to bind into the MHCII cleft, followed by migration to the cell membrane and presentation to another immune cell. Further in-cell studies are necessary to tackle this possibility, to answer whether aggregation of antigenic peptides would be a useful physiological strategy.

The tertiary structure of gp43 is not yet known, but due to its high sequence similarity with β-glucanase from other fungi, it was possible to generate a structural model (Fig. 1b). P10 seems to be solvent exposed in gp43, which makes sense since P10 is an effective epitope of Pb. Furthermore, a comparison between the primary sequence of P10 in gp43 (QTL**I**AI**HT**LA**I**RYA**N**) and that of the homologous region in β-glucanase from *Paracoccidioides lutzii* (QTL**A**AI**RA**LA**N**RYA**K**) reveals an important feature (the amino acids that differ between them are in bold), namely, the presence of two additional charges in the sequence of the enzyme (underlined, **R** and **K**), one in the middle and the other at the extreme of the C-terminal. Charged residues have gatekeeper properties that avoid the aggregation of peptides and proteins^28–30^. Interestingly, the corresponding stretch of P10 in β-glucanase has no tendency to aggregate as evidenced by bioinformatic analyses (Fig. 2b). Besides, P10 in β-glucanase lacks the HTLAIR sequence known to be the antigenic region^40,41^, which precludes its use as a vaccine candidate against Pb.

The sequence of P10 was established randomly by dividing gp43 into small peptides, which were further synthesized and tested for their immunogenic potential^10^. P10 was extremely potent in inducing a consistent immune response in animal models^48^ by promoting a Th-1 lymphocyte response and the production of IFN-γ, also protecting the animals against further fungal infection^10^. Later, using the algorithm TEPITOPE, which identifies immunodominant human T-cell epitopes of gp43^49^, several different peptides were predicted to bind with high affinity to multiple HLA-DR molecules and P10 was the most promiscuous among them^11^, binding to 84% of the antigens. This suggests that gp43 could be processed by immune cells that expose antigenic regions of gp43.

In order to get evidence regarding the formation of P10 or P10-like peptides by proteolysis of gp43, we took advantage of in-silico tools for prediction of protease cleavage products (Table 1). We agree that this is a first approach to gain insight into the formation of these peptides, since we do not have gp43 purified to perform this investigation in vitro. Interestingly, even by using these in-silico analyses it was shown that proteinase K, trypsin and pepsin could digest gp43 forming P10-like peptides (Table 1). We envision that the resident proteases of the endosome of immune cells would generate fragments of gp43, some of them spanning the P10 region. We analyzed those sequences with Aggrescan and found that most of them are very prone to aggregation.

We cannot rule out that P10 and P10-like peptides (Table 1), some of them with significant aggregation propensities, would be generated outside the cell, since gp43 is secreted by the fungi during growth, allowing the encounter of gp43 with tissue-resident proteases. Besides, Pb has an aspartyl protease similar to pepsin^50^, one of the proteases able to cleave gp43, generating P10-like peptides. These data suggest that these aggregation-prone fragments of gp43 may be formed under physiological conditions outside the cells. In this context, it is possible that fibrils of P10 and P10-like peptides contribute to Pb biofilm stabilization, as reported for several other biofilms in which amyloid fibrils act as biofilm matrix scaffolds^51,52^. Several of these fibrils are formed by proteolytic fragmentation of adhesins such as gp43 secreted by the pathogens^52^. Further studies are necessary to explore this possibility.

In the context of vaccine production, what can we learn from P10 like-peptides that display lower aggregation propensities than P10 yet still bear the antigenic HTLAIR sequence? In Table 2 there is list of peptides derived from P10 that could be useful for vaccine development against Pb. The rationale behind their sequences is explained in the Table. As seen, insertion of more than one charge, especially negative charges, at the N-terminal contributed more effectively at reducing P10 aggregation propensity.

**Table 2:**
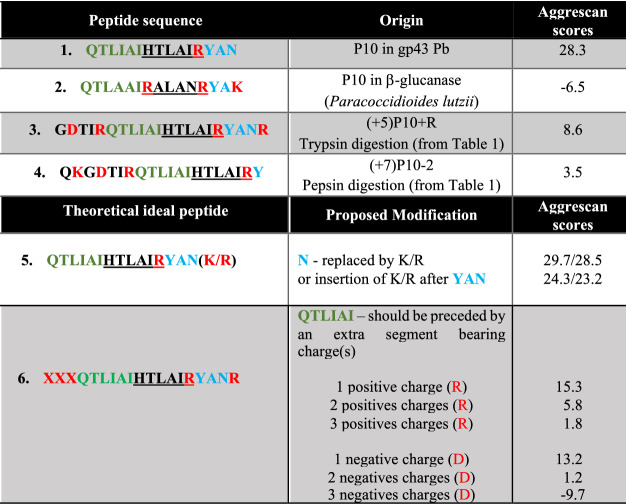
Rational design of P10-like peptides which might be explored as candidates for vaccine development.

Charged residues are in red. HTLAIR is the antigenic sequence, which was kept in all proposed sequences (peptides 5 and 6). This sequence is absent in P10 from *P. lutzii* (peptide 2). Note that insertion of positive charges at the C-terminal end of P10 (peptides 5) does not significantly change its aggregation propensity, while modifications at the N-terminal are more effective (peptides 6).

Taken together, this study aims to contribute to the design of candidate peptides for vaccine development in general and against Pb in particular, providing evidence for the need to study their aggregation propensity in vitro or even in silico to aid in the development of new effective vaccines. In addition, the possibility of obtaining aggregate-prone peptides derived from secreted pathogen proteins opens new possibilities for studying their functions in this aggregated state, a property displayed by functional amyloids^53^, which are amyloid with biological relevance. More studies are necessary to unravel whether antigen processing in endosome compartments in the immune cells also takes advantage of the aggregation properties of antigenic peptides such as P10 for storage, protection and release mechanisms. Besides, the possibility that peptides in an amyloidal conformation (with conformational epitopes stabilized) could elicit an immune response is another field of future investigation.

## Methods

### Gp43 secondary-structure prediction

Gp43 secondary-structure prediction was performed using PSIPRED software (19, http://bioinf.cs.ucl.ac.uk/psipred/). PSIPRED is an accurate secondary-structure prediction method that uses BLAST (Basic Local Alignment Search Tool) to find regions of local similarity between known homologous sequences. Gp43 primary sequence (GenBank: AAG36697.1) was used as input for the analyses using standard parameters and the webserver retrieved as output the most probable conformation (α-helix, β-sheet or random coil) of each residue in the sequence (Fig. 1a).

### Prediction of solvent accessibility

The solvent accessibility of gp43 residues was predicted by NetSurfP server, available at: http://www.cbs.dtu.dk/services/NetSurfP-2.0/. NetSurfP is a web-based server with a user-friendly interface able to predict solvent accessibility based on sequence. It uses an architecture composed of convolutional and long short-term memory neural networks trained on large data sets of solved protein structures available on public data banks. The gp43 sequence in FASTA format was used as input to the prediction using default parameters.

### Calculation of GRAVY (grand average of hydropathicity)

The GRAVY of peptides was calculated using the Expasy ProtParam toolkit available at: https://web.expasy.org/protparam/. ProtParam computes various physicochemical properties that can be deduced from a protein sequence. No additional information is required about the protein under consideration. Peptide sequences in FASTA format were used as input for the calculations. The GRAVY value for a peptide or protein is calculated as the sum of hydropathy values of all the amino acids, divided by the number of residues in the sequence.

### Generation of gp43 3D structural models

The recently released program AlphaFold^21^ is considered the first computational method that can predict protein structures with atomic accuracy even in the absence a known homologous structure. AlphaFold is based in a machine learning approach that incorporates physical and biological knowledge about protein structure, leveraging multi-sequence alignments, into the design of the deep learning algorithm. The program was validated in the 14th edition of the Critical Assessment of protein Structure Prediction (CASP14), demonstrating accuracy competitive with experimental structures. The full version of the software is found at https://alphafold.ebi.ac.uk, but it still works only as a data bank of already predicted structures. In our predictions, we used a version of AlphaFold available at https://colab.research.google.com/AlphaFold.ipynb that allows single predictions. We used as the input for the modeling, the sequence of gp43 retrieved from the Uniprot under the code Q9HER5_PARBR. The PDB file output from AlphaFold was visualized, edited and colored with PyMOL (The PyMOL Molecular Graphics System, Version 2.0 Schrödinger, LLC) to generate the models presented in Fig. 1.

We also generated a gp43 structure model using RaptorX webserver (22, http://raptorx.uchicago.edu/). RaptorX is a protein structure prediction server that excels at predicting 3D structures for protein sequences when protein homologs with homology degree > 30% are available in the Protein Data Bank (PDB). RaptorX allows for template-based tertiary structure modeling and delivers high-quality structural models. As the template for the modeling, the high-resolution structure of β-glucanase (sequence similarity > 80%) (PDB1CZ1) was used.

### In-silico analyses of aggregation propensity and the identification of amyloid- prone regions (APRs) in gp43

The intrinsic aggregation and amyloid formation propensities were evaluated either in the context of the complete gp43 protein sequence or considering the isolated derived peptides. These analyses were performed with AGGRESCAN^27,54^, employing default settings. ZipperDB^32,33^ was used to detect the presence of APRs capable of forming steric zippers in the sequences of P4, P10 and P23 peptides, also employing default settings. Primary sequence of gp43 P10-like structure from *Paracoccidioides lutzii* glucan 1,3-β-glucosidase also used in the analysis was obtained from UNIPROT code C1H4T0.

We further analyzed P4, P10 and P23 aggregation propensities using AmylPred2. AmylPred2 found at http://thalis.biol.uoa.gr/AMYLPRED2/ employs a consensus of different methods developed to predict features related to the formation of amyloid fibrils. The consensus of these methods is defined as the hit overlap of at least n/2 (rounded down) out of n selected methods (i.e. 5 out of 11 methods, if the user chooses to use all the 11 available methods)^31^. We used as input for the predictions the primary sequences of P4, P10 and P23 running the program with the default parameters in the webserver.

### In-silico models of amyloid fibril cores

The recently developed program Cordax^35^; https://cordax.switchlab.org/) was used to generate models of the amyloid cores (as steric zippers). The sequences of P4, P10 and P23 were analyzed using default settings. Cordax uses a large set of amyloid-core structures with atomic resolution deposited in the PDB as templates for the modeling approach. Briefly, to generate models of a query sequence, the program divides it into hexapeptides as the unit for the predictions (as in previously developed prediction methods). The side chains of the hexapeptides are modeled into all template structures of the library using the FoldX force field^55^. FoldX yields a model and an associated free-energy estimate of the fitting (ΔG, kcal/mol). Cordax gives two main outputs: i. the prediction of whether the segment is an amyloid- core sequence and ii. the most likely amyloid-core model of that segment (the structural topology, orientation of β-strands and overall architecture of the resulting putative fibril core). The output of PDB files from Cordax were visualized, edited and colored to generate the structures in Fig. 3 using PyMOL.

### Gp43 in-silico digestion by proteases

In-silico gp43 digestion was performed using ExpasyPeptideCutter^39^ to simulate the generation of cleavage products by different proteases. Enzymes predicted products of cleavage generated by the software were used to generate the data described in Table 1 as P10-like peptides, namely, peptides which include P10 sequence added or deleted by few residues at the C- or N-terminals.

### Peptides

Peptides purchased from BIOMATIK with purity above 95% were P4 (NLGRDAKRHLSKHWDTFITEDDFKNIAAAGL), P10 (QTLIAIHTLAIRYAN) and P23 (AFEVGAGWYFWTWKTEGAPGWDMQD). These were diluted in DMSO 100% to a final concentration of 5 mM (stock solution).

### Circular dichroism (CD) assays

Peptide samples were prepared by diluting stock solutions immediately before measurements to a concentration of 100 μM in 2,2,2-trifluoroethanol (TFE) 5% (soluble samples) or in PBS (aggregated samples). CD spectra were recorded at the indicated times of incubation, scanning from 260 to 190 nm on a Jasco 810 spectropolarimeter equilibrated at 25 °C. Each spectrum shown is the accumulation of 10 scans.

### In-vitro aggregation assays

For *in-vitro* aggregation, P4, P10 and P23 stock solutions were diluted in PBS under agitation in the presence of Thioflavin-T (ThT; 50 μM), a specific fluorescent probe for amyloid fibrils. ThT fluorescence emission was measured at 485 nm by exciting the samples at 450 nm. To measure light scattering (LS), samples were excited at 320 nm while emission was collected at 320 nm. For the seeding assays, a solution containing 100 µM previously formed P10 amyloid fibrils was sonicated (40 kHz) during 30 min for mechanical fibril fragmentation. Five μL of a seed suspension were added to a freshly prepared P4 peptide solution at 100 μM under agitation. Seeds were used at the final concentration of 5% unless otherwise stated.

### Mass spectrometry

P4-amyloid fibrils (formed in the presence of 5% P10 seeds) were centrifuged at 21,952×*g* for 30 min. After centrifugation, the supernatant was removed carefully, and the pellet was resuspended in 50 μL of a 9 M urea solution and incubated overnight under agitation. After that, the sample was diluted to 1 μM in 3% acetonitrile Wnano Acquity system (Waters, Milford, MA) for mass spectrometry. Proteins were desalted online using a trap column (Waters Symmetry C18, 180 μm × 20 mm, 5 μm) for 5 min and the liquid chromatography was performed with 3% to 85% acetonitrile containing 0.1% formic acid, 0.5 μL/min flow, in a HSS T3 130 C_18_ 100 μm × 100 mm, 1.7 μm analytical column (Waters, Milford, MA) for 58 min. System was set at initial conditions for 17 min to equilibrate the column.

Electrospray mass spectra were recorded using a Synapt HDMS quadrupole/orthogonal acceleration time-of-flight spectrometer (Waters, Milford, MA) interfaced to the nanoAcquity system. Capillary voltage was set at 3500 V, source temperature was 80 °C and cone voltage was 40 V. The instrument control and data acquisition were conducted by a MassLynx data system (Version 4.1, Waters), and experiments were performed by scanning mass-to-charge ratios (*m*/*z*) of 400–2000 using a scan time of 1 s, applied during the whole chromatographic process. A 320 fmol GFP – Glu Fibrino Peptide solution in 50% acetonitrile containing 0.1% formic solution was set at a 0.5 μL/min flow and acquired during 1 s after each 15 s of the main chromatogram to calibrate spectra using Q-Tof's LockSpray (Waters, Milford, MA).

All data were processed manually in MassLynx. To obtain an accurate molecular- mass measurement the resulting chromatographic peaks were analyzed, and the combined raw mass spectrum was lock-mass corrected in MS *m*/*z* scale using GFP ion 785.8426 m/z. Resulting spectra were treated using a charge-state deconvolution algorithm—Maximum entropy (MaxEnt 3, Waters, Milford, MA)—and monoisotopic singly charged ions were assigned in a relative intensity plot.

### Transmission electron microscopy (TEM)

Samples of aggregated peptides were diluted to 10 μM in Milli-Q water. Five μL of this suspension were absorbed onto 200-mesh carbon-coated copper grids for 5 min and then blotted to remove excess material. Negative staining was performed by adding 5 µL of 2% (w/v) uranyl acetate. Samples were dried in air for 3 min. The grids were imaged with a JEOL 1200 electron microscope (JEOL Ltd.) operating at a 60 kV acceleration voltage.

## Supplementary Information

Supplementary Figure S1.

## References

[1] Cordova L. A., Torres J. *Paracoccidioidomycosis in StatPearls*. (StatPearls Publishing, 2021). 33085335

[2] Sardi J (2015). In vitro *Paracoccidioides brasiliensis* biofilm and gene expression of adhesins and hydrolytic enzymes. Virulence.

[3] Braz JD (2021). Gene expression of Paracoccidioides virulence factors after interaction with macrophages and fibroblasts. Mem. Inst. Oswaldo Cruz..

[4] Vicentini AP (1994). Binding of *Paracoccidioides brasiliensis* to laminin through surface glycoprotein gp43 leads to enhancement of fungal pathogenesis. Infect. Immun..

[5] Mendes-Giannini MJ (2006). Binding of extracellular matrix proteins to *Paracoccidioides brasiliensis*. Microbes Infect..

[6] Cisalpino PS (1996). Cloning, characterization, and epitope expression of the major diagnostic antigen of *Paracoccidioides brasiliensis*. J. Biol. Chem..

[7] Marques da Silva, S. H. *et al*. Detection of circulating gp43 antigen in serum, cerebrospinal fluid, and bronchoalveolar lavage fluid of patients with paracoccidioidomycosis. *J. Clin. Microbiol.* **41**, 3675–3680. 10.1128/JCM.41.8.3675-3680.2003 (2003). 10.1128/JCM.41.8.3675-3680.2003 PMC179774 12904374

[8] Magalhães, A. *et al*. Prophylactic and therapeutic vaccination using dendritic cells primed with peptide 10 derived from the 43-kilodalton glycoprotein of *Paracoccidioides brasiliensis*. *CVI*. **19**, 23–29. 10.1128/CVI.05414-11 (2012). 10.1128/CVI.05414-11 PMC3255948 22089247

[9] Konno FT (2012). *Paracoccidioides brasiliensis* GP43-derived peptides are potent modulators of local and systemic inflammatory response. Microbes Infect..

[10] Taborda CP, Juliano MA, Puccia R, Franco M, Travassos LR (1998). Mapping of the T-cell epitope in the major 43-kilodalton glycoprotein of *Paracoccidioides brasiliensis* which induces a Th-1 response protective against fungal infection in BALB/c mice. Infect. Immun..

[11] Iwai, L. K. *et al.* In silico prediction of peptides binding to multiple HLA-DR molecules accurately identifies immunodominant epitopes from gp43 of *Paracoccidioides brasiliensis* frequently recognized in primary peripheral blood mononuclear cell responses from sensitized individuals. *Mol. Med. (Cambridge, Mass.)*. **9**, 209–219 (2003). PMC1430984 15208742

[12] Zhang L (2012). TEPITOPEpan: extending TEPITOPE for peptide binding prediction covering over 700 HLA-DR molecules. PLoS ONE.

[13] Mayorga O (2012). The role of adjuvants in therapeutic protection against paracoccidioidomycosis after immunization with the P10 peptide. Front. Microbiol..

[14] Rittner GM (2012). Therapeutic DNA vaccine encoding peptide P10 against experimental paracoccidioidomycosis. PLoS Negl. Trop. Dis..

[15] Sesardic D (1993). Synthetic peptide vaccines. J. Med. Microbiol..

[16] Li W, Joshi MD, Singhania S, Ramsey KH, Murthy AK (2014). Peptide vaccine: Progress and challenges. Vaccines.

[17] Eisenberg D, Jucker M (2012). The amyloid state of proteins in human diseases. Cell.

[18] Lacerda Pigosso, L. *et al*. *Paracoccidioides brasiliensis* presents metabolic reprogramming and secretes a serine proteinase during murine infection. *Virulence*. **8**, 1417–1434. 10.1080/21505594.2017.1355660 (2017). 10.1080/21505594.2017.1355660 PMC5711425 28704618

[19] Jones DT (1999). Protein secondary structure prediction based on position-specific scoring matrices. J. Mol. Biol..

[20] Cutfield SM (1999). The structure of the exo-beta-(1,3)-glucanase from *Candida albicans* in native and bound forms: Relationship between a pocket and groove in family 5 glycosyl hydrolases. J. Mol. Biol..

[21] Jumper J (2021). Highly accurate protein structure prediction with AlphaFold. Nature.

[22] Källberg M (2012). Template-based protein structure modeling using the RaptorX web server. Nat. Protoc..

[23] Ma J (2013). Protein threading using context-specific alignment potential. Bioinformatics (Oxford, England)..

[24] de Amorim J (2013). DNA vaccine encoding peptide P10 against experimental paracoccidioidomycosis induces long-term protection in presence of regulatory T cells. Microbes Infect..

[25] Klausen, M. S. *et al* NetSurfP-2.0: Improved prediction of protein structural features by integrated deep learning. *Proteins*. **87**, 520–527. 10.1002/prot.25674 (2019). 10.1002/prot.25674 30785653

[26] Soto C, Castaño EM (1996). The conformation of Alzheimer's beta peptide determines the rate of amyloid formation and its resistance to proteolysis. Biochem. J.

[27] Conchillo-Solé, O., de Groot, N. S., Avilés, F. X., Vendrell, J., Daura, X., & Ventura, S. AGGRESCAN: A server for the prediction and evaluation of "hot spots" of aggregation in polypeptides. *BMC Bioinform*. *8*. 10.1186/1471-2105-8-65 (2007). 10.1186/1471-2105-8-65 PMC1828741 17324296

[28] Monsellier E, Chiti F (2007). Prevention of amyloid-like aggregation as a driving force of protein evolution. EMBO Rep..

[29] Rousseau F, Serrano L, Schymkowitz JW (2006). How evolutionary pressure against protein aggregation shaped chaperone specificity. J. Mol. Biol..

[30] Sant'Anna R (2014). The importance of a gatekeeper residue on the aggregation of transthyretin: Implications for transthyretin-related amyloidoses. J. Biol. Chem..

[31] Tsolis A. C., Papandreou N. C., Iconomidou V. A. & Hamodrakas S. J. A consensus method for the prediction of 'aggregation-prone' peptides in globular proteins. *PLoS ONE*. **8**. 10.1371/journal.pone.0054175 (2013). 10.1371/journal.pone.0054175 PMC3542318 23326595

[32] Thompson MJ, Sievers SA, Karanicolas J, Ivanova MI, Baker D, Eisenberg D (2006). The 3D profile method for identifying fibril-forming segments of proteins. Proc. Natl. Acad. Sci..

[33] Nelson R (2005). Structure of the cross-beta spine of amyloid-like fibrils. Nature.

[34] Goldschmidt L, Teng PK, Riek R, Eisenberg D (2010). Identifying the amylome, proteins capable of forming amyloid-like fibrils. Proc. Natl. Acad. Sci. USA.

[35] Louros N, Orlando G, De Vleeschouwer M, Rousseau F, Schymkowitz J (2020). Structure-based machine-guided mapping of amyloid sequence space reveals uncharted sequence clusters with higher solubilities. Nat. Commun..

[36] Sabate R, Espargaro A, Graña-Montes R, Reverter D, Ventura S (2012). Native structure protects SUMO proteins from aggregation into amyloid fibrils. Biomacromol.

[37] Jarrett, J. T. & Lansbury P. T. Jr. Amyloid fibril formation requires a chemically discriminating nucleation event: studies of an amyloidogenic sequence from the bacterial protein OsmB. *Biochem.* **31**, 12345–52. 10.1021/bi00164a008 (1992). 10.1021/bi00164a008 1463722

[38] Harper JD, Lansbury PT (1997). Models of amyloid seeding in Alzheimer's disease and scrapie: Mechanistic truths and physiological consequences of the time-dependent solubility of amyloid proteins. Annu. Rev. Biochem..

[39] Gasteiger, E. et al. Protein identification and analysis tools on the ExPASy server. In *The Proteomics Protocols Handbook. Springer Protocols Handbooks* (ed. Walker, J. M.) 571–607 (Humana Press, 2005). 10.1385/1-59259-890-0:571.

[40] Morais FV, Barros TF, Fukada MK, Cisalpino PS, Puccia R (2000). Polymorphism in the gene coding for the immunodominant antigen gp43 from the pathogenic fungus *Paracoccidioides brasiliensis*. J. Clin. Microbiol..

[41] Travassos, L. R., Taborda, C. P., Iwai, L. K., Cunha-Neto, E. C. & Puccia, R. The gp43 from *Paracoccidioides brasiliensis*: A major diagnostic antigen and vaccine candidate in Human Fungal Pathogens (eds. Domer J. E. & Kobayashi G. S.). 10.1007/978-3-662-10380-7_15 (Springer, 2004).

[42] Grifoni A, Sidney J, Zhang Y, Scheuermann RH, Peters B, Sette A (2020). A sequence homology and bioinformatic approach can predict candidate targets for immune responses to SARS-CoV-2. Cell Host Microbe.

[43] Lal H (2015). Efficacy of an adjuvanted herpes zoster subunit vaccine in older adults. N. Engl. J. Med..

[44] Richmond P (2021). Safety and immunogenicity of S-Trimer (SCB-2019), a protein subunit vaccine candidate for COVID-19 in healthy adults: A phase 1, randomised, double-blind, placebo-controlled trial. Lancet.

[45] Rappuoli, R., Bottomley, M. J., D'Oro, U., Finco, O. & De Gregorio, E. Reverse vaccinology 2.0: Human immunology instructs vaccine antigen design. *J. Exp. Med*. **213**, 469–481. 10.1084/jem.20151960 (2016). 10.1084/jem.20151960 PMC4821650 27022144

[46] Fabris, V. E. Amiloidose experimental no hamster (*Mesocricetus auratus*) induzida pelo *Paracoccidioides brasiliensis*: aspectos histologicos e ultraestruturais do rim; estudo da função renal, eletroferese e imunoeletroforese das proteinas sericas e urinarias. 1976. 99 f. Tese (doutorado) - Universidade Estadual de Campinas, Faculdade de Ciencias Médicas, Campinas, SP. http://www.repositorio.unicamp.br/handle/REPOSIP/310075. Accessed 14th July 2018.

[47] Zepeda-Cervantes J, Vaca L (2018). Induction of adaptive immune response by self-aggregating peptides. Expert Rev. Vaccines.

[48] Silva L, Dias LS, Rittner G, Muñoz JE, Souza A, Nosanchuk JD, Travassos LR, Taborda CP (2017). Dendritic cells primed with *Paracoccidioides brasiliensis* peptide P10 are therapeutic in immunosuppressed mice with paracoccidioidomycosis. Front. Microbiol..

[49] Nielsen M, Lund O, Buus S, Lundegaard C (2010). MHC class II epitope predictive algorithms. Immunology.

[50] Tacco BA (2009). Characterization of a secreted aspartyl protease of the fungal pathogen *Paracoccidioides brasiliensis*. Med. Mycol..

[51] Schwartz, K., Syed, A. K., Stephenson, R. E., Rickard, A. H. & Boles, B. R. Functional amyloids composed of phenol soluble modulins stabilize *Staphylococcus aureus* biofilms. *PLoS Pathog*. **8**. 10.1371/journal.ppat.1002744 (2012). 10.1371/journal.ppat.1002744 PMC3369951 22685403

[52] Taglialegna A, Lasa I, Valle J (2016). Amyloid structures as biofilm matrix scaffolds. J. Bacteriol..

[53] Maji SK (2009). Functional amyloids as natural storage of peptide hormones in pituitary secretory granules. Science.

[54] Sánchez de Groot, N., Pallarés, I., Avilés, F. X., Vendrell, J. & Ventura, S. Prediction of “hot spots” of aggregation in disease-linked polypeptides. *BMC Struct. Biol.* **30**. 10.1186/1472-6807-5-18 (2005). 10.1186/1472-6807-5-18 PMC1262731 16197548

[55] Guerois R, Nielsen JE, Serrano L (2002). Predicting changes in the stability of proteins and protein complexes: A study of more than 1000 mutations. J. Mol. Biol..

