# Supplementary material for: Peptides derived from gp43, the most antigenic protein from Paracoccidioides brasiliensis, form amyloid fibrils in vitro: implications for vaccine development
Source: Sci Rep. 2021 Dec 6;11:23440. doi: 10.1038/s41598-021-02898-5 (PMC8648789; doi:10.1038/s41598-021-02898-5)
Supplement: Supplementary file 1 — Supplementary Figure S1. [file 41598_2021_2898_MOESM1_ESM.docx]

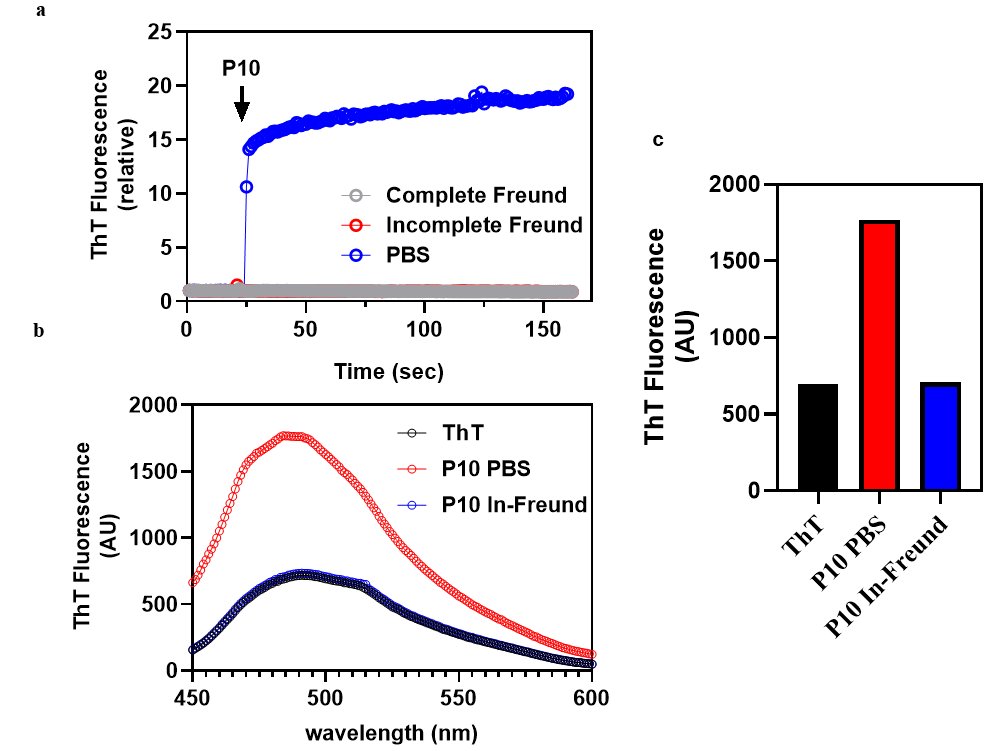


**Supplementary Figure 1 – P10 does not aggregate in vaccine adjuvants.** 100 μM of P10 were incubated in PBS (blue), complete (grey) or incomplete (red) Freund’s adjuvant containing thioflavin-T (ThT) to measure amyloid formation. **(B)** After aggregation kinetics, solutions were centrifuged and resuspended in a PBS-Thioflavin solution and ThT fluorescence emission spectra were recorded, confirming that fibrils were formed only in PBS. **(C)** P10 was aggregated at pH 7.5, 25 ^o^C in the absence of ThT and after 60 min, ThT was added and its emission was recorded to evaluate fibril formation.
